# Using Radiology images to characterize an angle of insertion for S1/S2 and SI joint injections: A retrospective observational study

**DOI:** 10.1016/j.inpm.2022.100068

**Published:** 2022-02-12

**Authors:** Jason L. Chang, Ajay K. Patel, Devas J. Modi, Peyton Johnson, Taif Mukhdomi, Amitabh Gulati

**Affiliations:** a Department of Rehabilitation & Regenerative Medicine, NewYork-Presbyterian Hospital- University Hospital of Columbia and Cornell, New York, NY, USA; b Department of Anesthesiology, NewYork-Presbyterian Hospital-Weill Cornell Medicine, New York, NY, USA; c Department of Anesthesiology and Critical Care, Memorial Sloan Kettering Cancer Center, New York, NY, USA

## Abstract

**Objective:**

Sacroiliac (SI) joint, S1 and S2 nerve root pathology are well documented in literature as common etiologies for low back pain. Evidence demonstrating starting angle of needle insertion during S1/S2 transforaminal and SI joint injections are lacking. Using computerized tomography (CT) radiography of the lumbosacral spine, this retrospective observational study seeks to characterize a starting angle of needle insertion at the sacral spine.

**Methods:**

This was a single-centered, retrospective observational study performed on adult cancer patients who had CT radiography of their lumbosacral spine, without significant sacral pathology, at our hospital from January 2016 and May 2021. For each patient, we determined the anatomical location of where the S1/S2 neural foramen and SI joint widens up. Using the annotation tools available in EIM image viewer, a maximum level and minimum level of insertion was recorded in order to calculate the average angle of insertion needed for procedural performance.

**Results:**

Through the analysis of 64 patients, average angle of insertion was 25° ​± ​1.36 for S1, 34° ​± ​1.93 for S2, and 33° ​± ​1.95 for SI injections. There were no statistically significant differences in angles when stratified based on laterality, gender, age, and BMI.

**Conclusion:**

Average angle of insertion to target the S1, S2 neural foramen and SI joint are 25° ipsilateral oblique, 34° ipsilateral oblique, and 33° contralateral oblique respectively starting with a squared sacral endplate. To our knowledge, there are no studies in the current literature that have attempted to identify an entry angle to target these anatomical structures.

**Six key words:**

Chronic Pain, SI Joint Pain, Steroid Injection, S1 Transforaminal, S2 Transforaminal, Needle Placement.

## 1 Introduction

Low back pain with and without radiculopathy is the global leader in years lived with disability (YLD) and accounts for 64.9 million YLD worldwide [1,2]. Sacroiliac (SI) joint, S1 and S2 nerve root pathology are well documented in literature as common etiologies [[3], [4], [5], [6]]. Symptoms include localized and radicular pain, paresthesias and weakness of the lower extremities.

Management of SI joint pain and S1/S2 radicular pain often includes oral medications, physical therapy, manual manipulation, invasive stabilization, radiofrequency (RF) denervation procedures, as well as analgesic and steroid injections [7,8]. Among these options intraarticular injections for SI joint as well as transforaminal injections for S1 and S2 nerve roots are effective and popular minimally invasive treatment selections [3,6,9]. Common guidance options include ultrasound, computerized tomography (CT) and fluoroscopy. Despite the prevalent use of these modalities there remains a paucity of evidence for the starting angle of a needle for SI joint, S1 and S2 transforaminal injections.

The objective of this retrospective observational study is to utilize CT image angle measurements to describe injection trajectories and correlate them to SI joint and S1/S2 transforaminal injections. If knowledge of more homogenous parameters for angle insertion can be found, this information may be of benefit to interventionalists worldwide for improved pre-procedural planning, enhanced execution of injection, decreased procedural time, and increased patient satisfaction.

## 2 Methods

### 2.1 Data sources

PubMed, PubMed Central, and Google Scholar were the primary sources for literature review for this manuscript.

### 2.2 Study design

This study was approved by the Institutional Review Board of Memorial Sloan Kettering Cancer Center and was supported by the Department of Anesthesiology and Critical Care (NIH Core Grant P30). The study was granted waiver of informed consent because it evaluated existing records, was not greater than minimal risk, and was deemed to be Health Insurance Portability and Accountability Act compliant because safeguards were in place to protect the personal health information of the subjects. This is a single-centered, retrospective observational study performed on adult cancer patients at Memorial Sloan Kettering Cancer Center who had a computed tomography scan of their total spine or lumbosacral spine at our hospital from January 2016 and May 2021.

### 2.3 Study population

We used an existing database consisting of a total of 160 patients with CT spine radiography. Demographic information including age, sex, weight, height, BMI, and cancer diagnosis were obtained by chart review (See Table 1). Medical imaging records were reviewed and the most recent lumbar or total spine CT scan were used for radiographic analysis. Patients with any sort of sacral or iliac pathology, instrumentation, primary or metastatic disease were excluded from the study. Patients with lumbar instrumentation, pathology, or disease were not excluded. Of the original 160 patients available in the database, sixty-four were included in the final analysis after meeting the selection criteria. This included 35 males and 29 females.

**Table 1:** **Patient demographics**.

	Study Population
Sample size, n	64
Sociodemographic Characteristics	
Mean age (SD) in years	64 (14.1))
Sex, n (%)	
Male	35 (55%)
Female	29 (45%)
Mean height (SD) in centimeters	165.5 (9.3)
Mean weight (SD) in kilograms	75.6 (20)

Abbreviations: SD, standard deviation.

### 2.4 Data collection

**Fig. 1:**
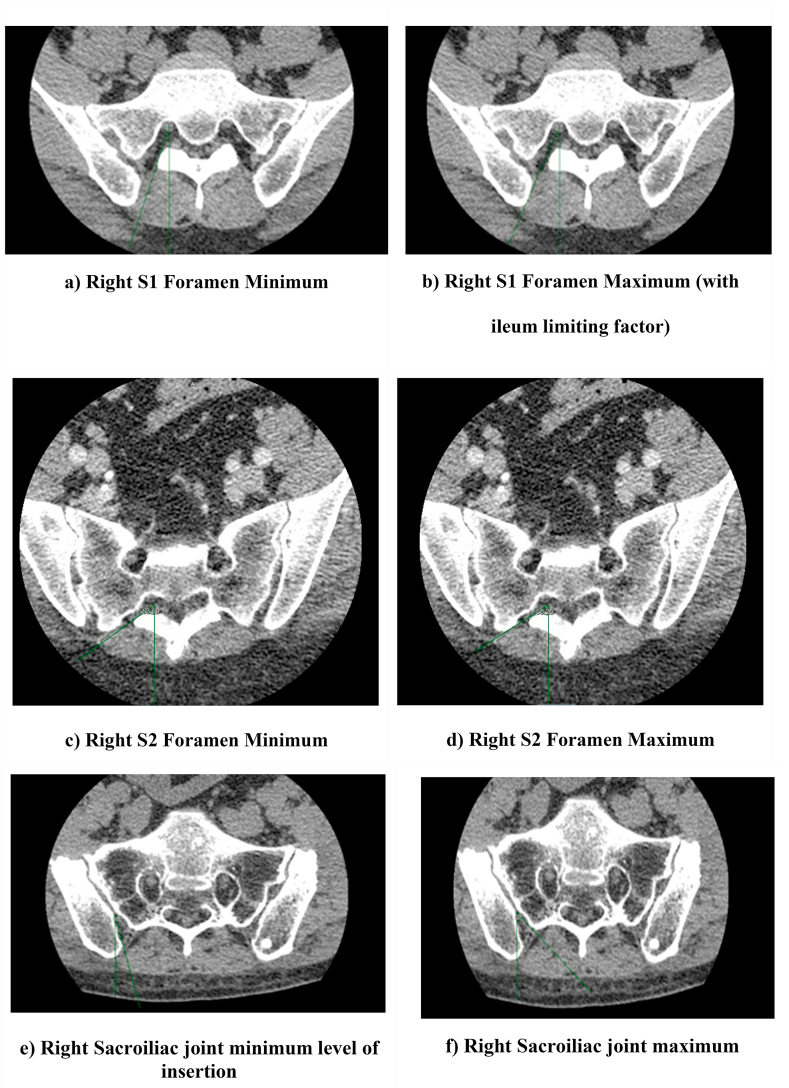
**Data Collection Method.** 1) A line was drawn perpendicular to the skin from the center of the nerve root (S1/S2) or the deepest part of the SI joint. 2) A minimum and maximal line tangential to the bony structures that surround the opening drawn 3) Minimum and maximal angles measures using angle tool.

The database of 160 patients were divided amongst 4 reviewers (AP, JC, DM, PJ). Each individual reviewed the chart to ensure the patient met inclusion criteria. Subsequently, images were accessed using the EIM image viewer, in which the most recent total spine or lumbosacral CT scan date was analyzed. For each patient, axial images were first analyzed to the point where the S1 foramen was noted to have the largest opening. At this level, the imaging “slice” was recorded. Using the annotation tools available in EIM image viewer, a maximum level and minimum level of insertion was recorded. This angle was measured by drawing a perpendicular line from the middle of the S1 nerve root to the posterior surface. An oblique line was drawn along the line of the S1 foramen, measuring both the maximum and minimal angles possible for insertion. Examples are provided in Fig. 1. The sacral shape (conical vs flat) and limiting factor (sacrum vs ileum) affecting the maximal angle of insertion was recorded. The same process was followed for bilateral S1 foramen and S2 foramen. These image slices correlate with the fluoroscopic images when the sacral endplates at L5/S1 are lined up and spinous processes are midline.

The angle of insertion for the sacroiliac joint was analyzed using cross-sectional views. The largest opening of the sacroiliac joint was first identified at either above the S1 foramen, upper-half between S1–S2, lower half between S1–S2, or below S2. After identification, a perpendicular line to the surface of the skin was drawn from the deepest part of the SI joint at point of inflection (See Fig. 1). Similar to the S1 and S2 foramen, an oblique line was drawn along the sacroiliac joint to determine both the minimum and maximum angle of insertion. The sacral shape (conical vs flat) was also recorded.

### 2.5 Statistical analysis

Patient demographic information was collected and recorded in Microsoft Excel 2016. We utilized the mean formula to determine the average age, weight, height, and BMI of the included study population. After bilateral S1, S2, and Sacroiliac joint minimum and maximum angles of insertion were recorded, we calculated the mean, min, and max angle of insertion for the aforementioned foramina and joint opening. A z score of 1.96 was utilized to represent the 95% confidence interval. Using the mean, standard deviation (SD), and confidence coefficient of 1.96 we were able to calculate the margin of error (MOE) and confidence interval for S1, S2 and SIJ. The percent of patients from our study that had lumbar pathology was calculated. In addition, the limiting factor for S1 and S2 entry, and location of largest sacroiliac joint opening was categorized. We then further stratified our data based on laterality (left vs. right) for each sacral level, gender, age, and BMI.

## 3 Results

**Fig. 2:**
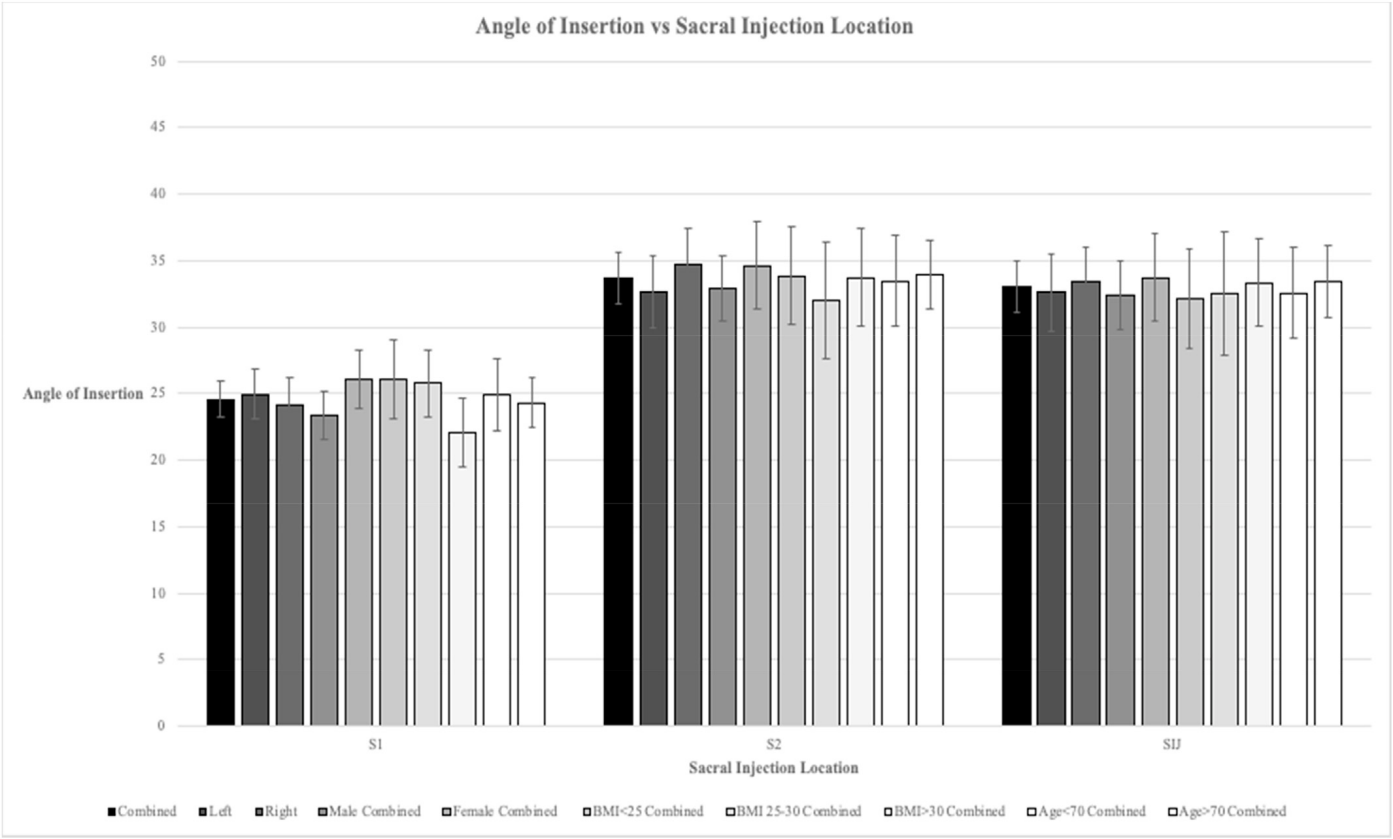
**Angle of Insertion vs. Sacral Injection Location**.

**Fig. 3:**
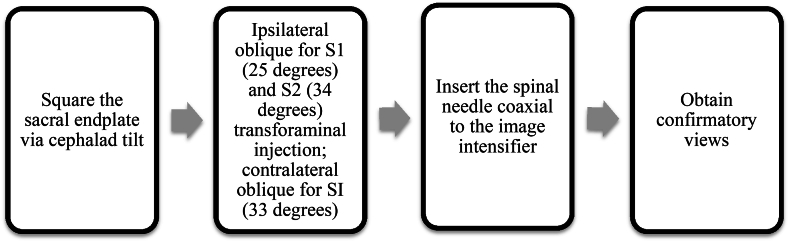
**Procedural protocol using** characterized **angles of insertion**.

Sixty-four total patients were analyzed, consisting of 35 males and 29 females. The average age of the studied population was sixty-four years old, weight 75.62 ​kg, height 165 ​cm, and BMI 27.5. The average angle of insertion for S1 was a minimum of 19° and a maximum of 30°. The average angle of insertion for S1 was 25° ​± ​1.36 95% CI [23.22, 25.96]. The average angle of insertion for S2 was a minimum of 23° and a maximum of 44°. The average angle of insertion for S2 was 34° ​± ​1.93 95% CI [31.79, 35.65]. The average angle of insertion for SI was a minimum of 21° and a maximum of 44°. The average angle of insertion for SI was 33° ​± ​1.95 [31.09, 35.00]. When separated based on laterality, averages were rather similar between the right and the left and the confidence intervals overlapped. There were no statistically significant differences in terms of the angle at each sacral level and SI joint when stratified based on gender, BMI (normal weight, overweight, and obese), and age (younger than 70 years old versus older than 70 years old). See Fig. 2 (see Fig. 3).

The limiting factor for S1 entry is mostly the ilium on both the right and left side, 89% of patients and 84% patients respectively. It seems to correlate with a flat sacral shape 77% of the time on the right and 74% on the left. The limiting factor for S2 entry is mostly the sacrum on the right and left side, 92% of patients and 94% of patients respectively. Also, it was found that about half the patients had the largest SI joint opening was at the upper half between the distance from S1 to S2, 53 patients, and the other half had the largest opening at the lower half between the distance from S1 to S2, 53 patients, and two patients had their largest SI joint opening below S2. 83% of patients had the largest SI opening at the same relative location on both the right and left side.

## 4 Discussion

**Fig. 4:**
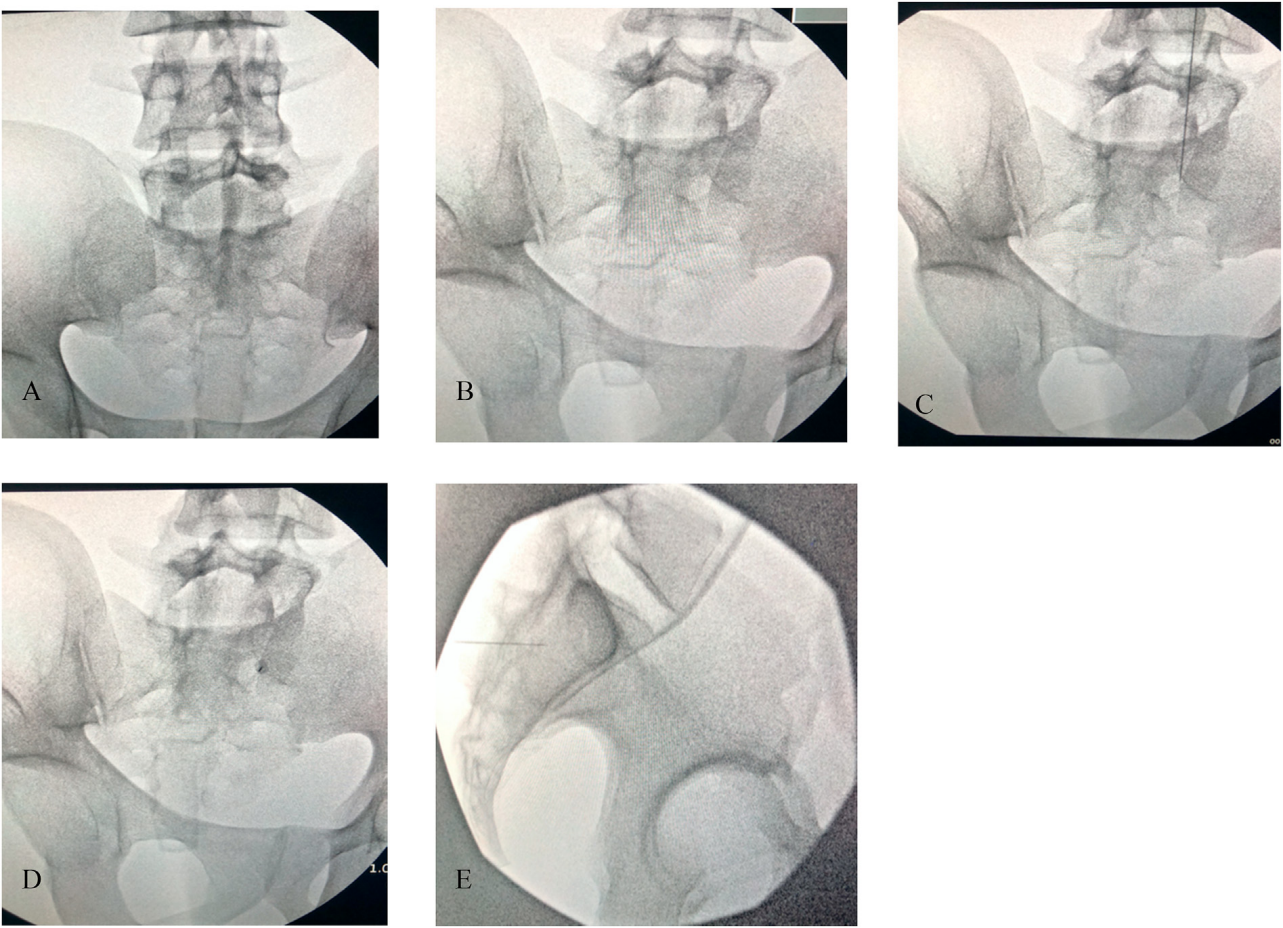
**Demonstration of S1 Transforaminal** Injection **on BioTras Model.** A demonstration of 25° ipsilateral oblique for S1 transforaminal injection on BioTras model. A) Squared sacral endplate B) Ipsilateral oblique 25° to open up Right S1 foramen. C) Marking the lateral edge of the Right S1 foramen with needle. D) Co-axial insertion with the C-arm at 25° ipsilateral. Advance needle co-axially until inside foramen. E) Lateral view for confirmation.

**Fig. 5:**
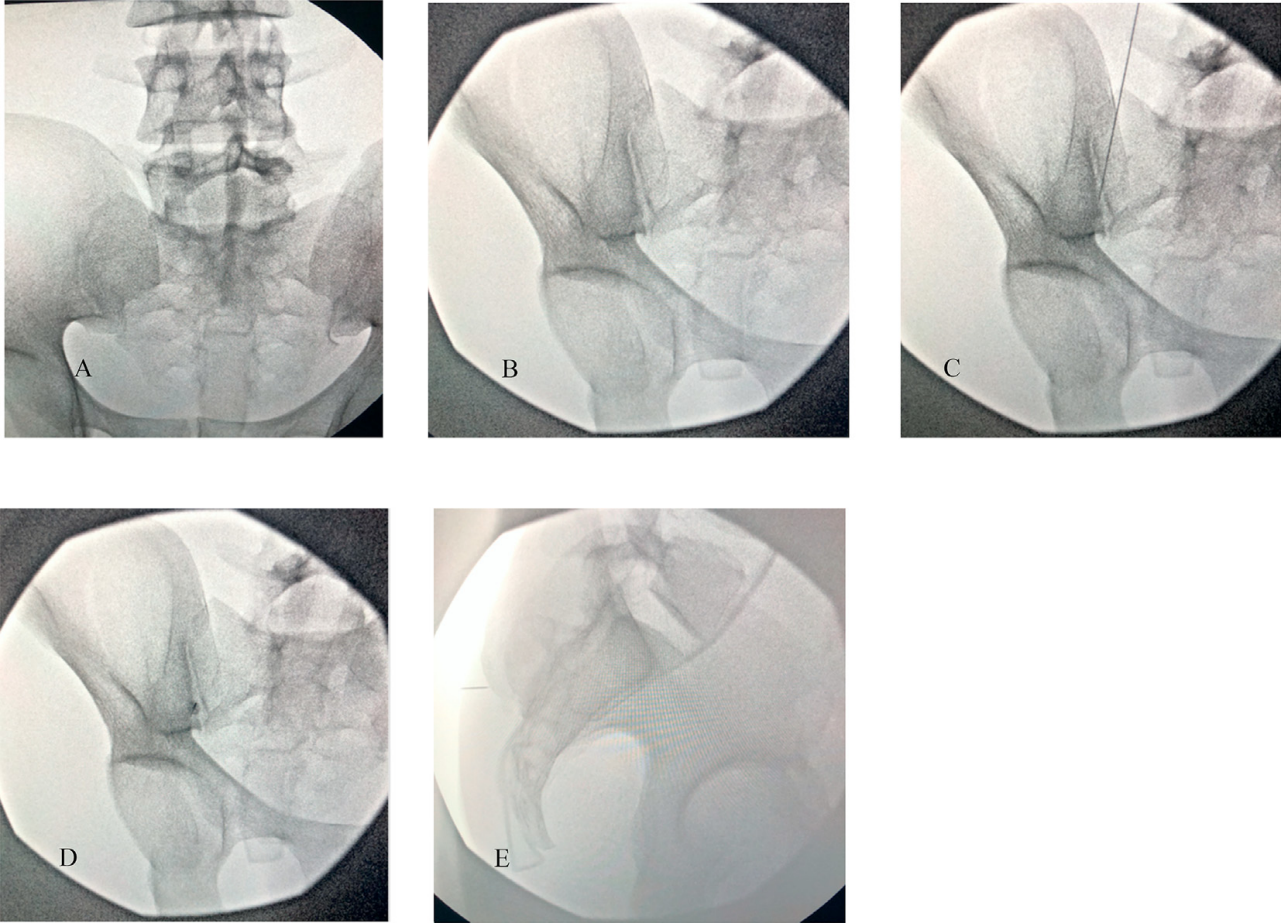

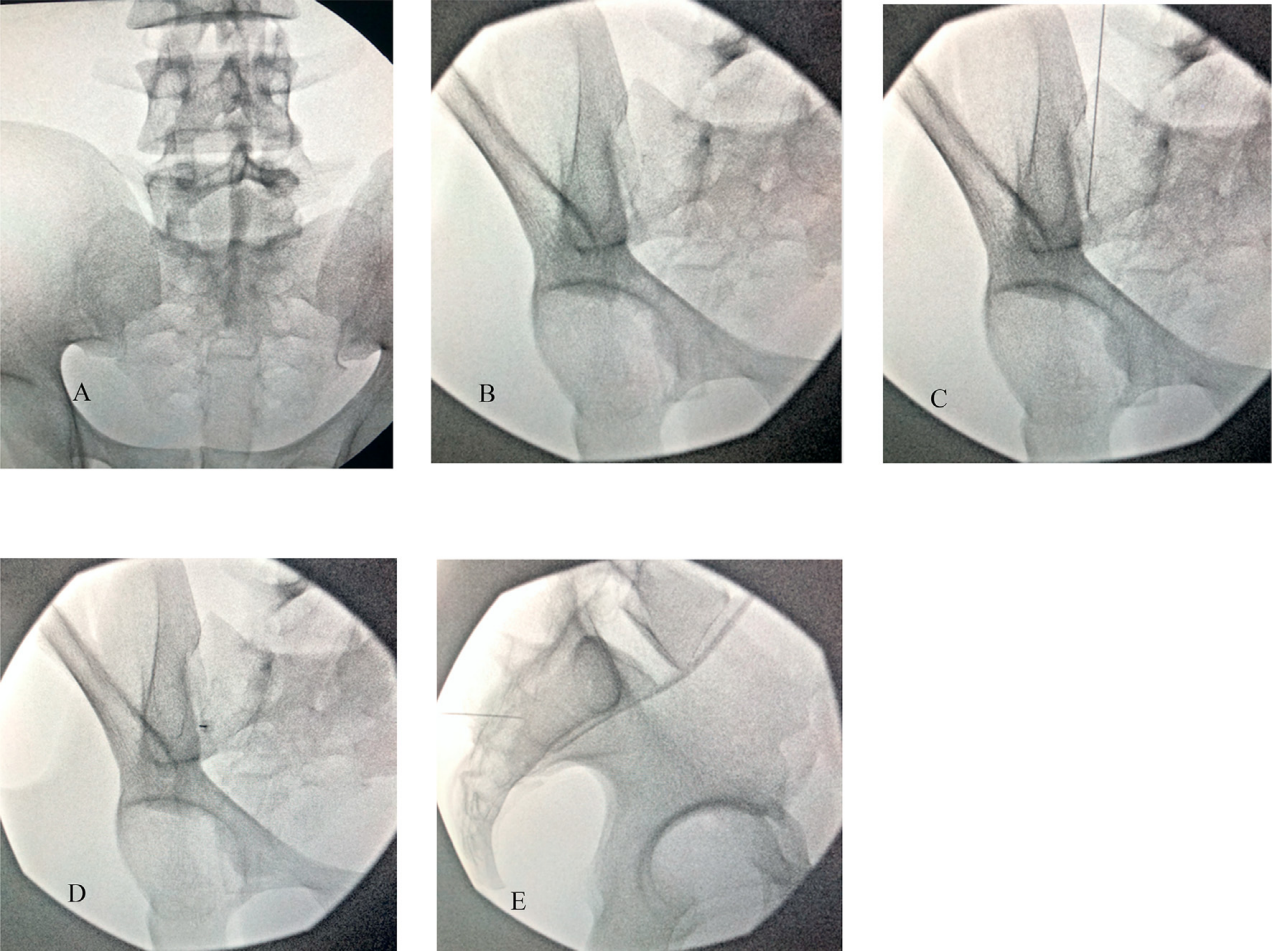
**I: Demonstration of Traditional SI Joint Injection on BioTras Model.** Demonstration of current practice of SI joint injections on BioTras model using the standard 20° contralateral oblique. A) squared sacral endplate. B) 20° contralateral oblique C-arm. C) Marking insertion site with needle. D) Co-axial insertion of the needle. E) Advance needle but impeded by ilium, demonstrating that even with seemingly open joint, current standard recommended angle does not fully open up the SI joint. **II: Demonstration of Calculated SI Joint Injection on BioTras Model.** Demonstration of utilization of our recommended angle of 33° contralateral oblique for SI joint injection improves opening SI joint in BioTras model. A) Squared sacral endplate. B) 33° contralateral oblique. C) Marking insertion site with needle. D) Co-axial insertion of the needle. E) Advance needle until inside SI joint with confirmatory lateral view.

The major finding of the retrospective observational study was that the average angle of insertion for S1 was 25° ​± ​1.36, S2 was 34° ​± ​1.93, and SI was 33° ​± ​1.95 oblique tilt. These angles of insertion correlate with CT fluoroscopy during procedural practice when the L5/S1 sacral endplate is squared and the spinous processes are midline. This starting view is crucial to have a standardized approach for angle guidance and translatability to real world practice under fluoroscopy. This is demonstrated in Fig. 4 where S1 foramen is easily accessed with a co-axial insertion of 25° ipsilateral oblique after L5/S1 sacral endplate squaring (see Fig. 5).

Current protocols based on Furman et al. transforaminal S1 epidural steroid injections involved lining up the superior S1/S2 endplate with a cephalad tilt and then subsequent slight ipsilateral oblique view [10]. The interventionist advances the needle co-axially and walk off the sacral periosteum around the dorsal S1/S2 foramen. For SI joint injections, they should be performed with a cephalad tilt of approximately 10–15° to elongate the posterior plane of the joint. A 10–20° contralateral oblique subsequently opens up the joint space. A practitioner then targets the needle 1–2 ​cm superior to the inferior aspect of the medial joint space coaxially with the C-arm. Intra-articular access via the inferior approach may not always be possible because of the anatomic complexity of the joint, or when it is affected by ankylosis or osteoarthritis. Hence, a superior approach can be considered.

Our recommended angles differ from current practices where they only offer a slight oblique tilt for the sacral transforaminal injections and only 10–20° contralateral oblique tilt for SI joint injections. This comes to show that the current recommended protocoled angles are likely not opening the joints or allowing for full visualization of the target structure. Fig. 5-I shows that despite seemingly open SI joint at the recommended upper limit of the contralateral oblique angle tilt (20°) with good visualization of the SI joint, co-axial insertion results in impedance by the ilium, which is not seen in Fig. 5-II. Thus, our preliminary data demonstrates a larger entry angle (which equates to a more medial to lateral approach) is important to enter the sacroiliac joint when compared to the currently accepted technique. If practitioners utilized our recommended angles, there could be potentially improved procedural accuracy, which needs to be further studied in clinical practice.

For S1, the limiting factor is mostly the ilium because of a flat sacral shape. Because of this, the varying pelvis types in women may influence the angle. There are 4 main types of pelvis: gynecoid, platypelloid, android, and anthropoid [11]. Given the anatomy of the gynecoid pelvis shape with the high riding ilium, there is potential for a smaller angle of insertion with the anatomical limitation from the ilium. However, we did not find any statistical differences when we stratified the angles based on gender. Interestingly, the limiting factor for S2 entry is mostly the sacrum as the ilium tapers down in the lower sacral levels. In terms of the sacroiliac joint injection, there was an even split where around 50% of the patient had the largest SI joint opening in the upper half between the distance from S1 to S2 and the other 50% had the largest opening in the lower half between the distance from S1 to S2. This could explain why there is a huge range of locations practitioners inject for the SI joint ranging from the traditional inferior approach to now a more superior approach.

To further fine-tune the starting angles of insertion, we also investigated MRI imaging of the lumbar and sacral spine. We compared our CT angle measurements with that of MRI and found that the MRI angles of insertion vary anywhere from 0 to 10° or more. We believe that this difference is due to variability in the angle of the cuts in MRI and thickness of the cuts. Thus, given that the slices are different, there isn't an optimal way to compare the angles of MRI versus CT. We chose to utilize CT spine images because CT displays the bone window well, allowing for easier and more accurate measurements of the insertion angles. Furthermore, CT is a much better correlate to fluoroscopy than MRI as well.

Future studies may include larger patient sample size to increase the resolution of any difference in angles between sacral level, gender, age, and BMI. Further stratification based on pelvic shape can also provide more information in the contralateral oblique angle based on personalized anatomy. Lastly, the incorporation of more reviewers or machine learning algorithms to make measurements can also further reduce error and variability in angle measurements. Another option would be to 3D print the iliosacral spines from the CT images to obtain clear three-dimensional views to enable improved angle measurements for S1, S2, and SI joint injections.

## 5 Limitations

One of the limitations of the study is the small sample size of 64 patients. We initially started off with 160 patients with CT spine radiography, but we excluded patients with any sort of sacral or iliac pathology, instrumentation, primary or metastatic disease. This is so that these pathologies or instrumentation do not alter the angles that are collected so that the angles can be more generalizable. Patients with lumbar instrumentation, pathology, or disease were not excluded as this did not affect the sacral or SI joint anatomy. When we attempted to differentiate the angle measurements based on sex, age, and BMI did not yield any difference, however this could have been a result of a small sample size. We hope to utilize this initial data as a proof of concept for a larger pilot study to conclusively demonstrate these new angles should be adopted as standard of care for which we would require at least around 3000 patient study for adequate power.

Another limitation of our study is the level at which we measured the SI joint angle of insertion does not necessarily correlate with common clinical practice. Our angle was measured at the deepest point in the joint before inflection which is not necessary to administer an intra-articular SI joint injection. However, this would obtain a smaller range which could be wider in actual clinical practice. Traditionally, SI joint injections are performed at a more caudal location where the joint is noted to be more synovial in nature. Our measurements are based on CT images, ranging from the level of the PSIS down to S2, which is thought to be a more fibrous part of the joint, but upon our imaging review, had a wide range of access. These measurements could offer another access point for the SI joint which could be explored further in clinical practice.

For our S1 and S2 measurements, the angle of insertion was measured to a depth of the nerve root exiting the spinal canal. Based on clinical practice, we understand that a spinal needle does not necessarily need to be advanced to the nerve root as adequate epidural flow can be obtained from a more posterior point. However, by measuring to the point of the S1 or S2 nerve root, the angle of access is likely to be underestimated and therefore in clinical practice epidural access could be obtained at a wider range. The angles measured in our study are of greater obliquity than traditionally used in practice and could offer an improved view under fluoroscopy of the dorsal aperture.

Furthermore, although our study shows that the widest point of entry is 50% in the upper half and 50% in the lower half between S1 and S2 foramen, many times the interventionalist does not have advanced imaging of the pelvis and will not have information regarding which region of the SIJ to target. It may have been simpler if we measured the angle of approach for the dorsal joint opening at the inferior border of the SIJ (the traditional approach) for better standardization, but this methodology might not provide the location of easiest access as found in our study. Future studies can potentially study the correlation of detecting the largest SIJ opening with coronal images that are obtained during fluoroscopy with that obtained from axial cross sections on CT.

Other limitations include variations and human error in the measuring method utilized via the EIM imager viewer angle measuring tool. Each patient's imaging was reviewed separately by four independent reviewers for the collection of the data. Prior training of the evaluators on multiple samples and crossed-referencing with each other prior to collecting all the measurements to standardize the method and minimize this human error.

## 6 Conclusion

This retrospective observational study analyzed CT images of the lumbosacral spine to determine a standard angle to target the S1, S2 foramen and SI joint for steroid injections to treat axial low back pain. We found that the average angle of insertion to target the S1 and S2 foramen is most often 25 and 34° respectively and may be the best angle for visualizing the point of entry. For the SIJ, the widest point of entry is between S1 and S2 and has an average insertion angle of 33° oblique tilt. These angles of insertion differ from what is currently taught in textbooks and could potentially allow for better visualization of the foramen and SI joint if practitioners started using our new recommended angles. Some limitations include our small sample size, but we wanted to ensure our inclusion criteria allowed our results to be more generalizable to the normal population without sacroiliac pathology. There is a paucity of data regarding a starting position for needle insertion. It is our hope that the data presented in this study can guide interventionalists with a characterized angle of insertion for S1, S2 TFESI and SI joint injections to improve procedural efficiency, accuracy, and ultimately potential patient outcomes.

## 7 Contributors

AG is the guarantor. He developed the idea for the study, designed data collection tools, method of analysis, monitored data collection for the whole study, cleaned and analyzed the data, and assisted in drafting and revising the manuscript prior to submission. JC also assisted in creating data collection tools, performed data collection through imaging review, analyzed the data, assisted in drafting the manuscript, and approved the final version to be published. AP assisted in creating data collection tools, performed data collection through imaging review, analyzed the data, assisted in drafting the manuscript, and approved the final version to be published. DM assisted in creating data collection tools, performed data collection through imaging review, analyzed the data, assisted in drafting the manuscript, and approved the final version to be published. PJ assisted in creating data collection tools, performed data collection through imaging review, analyzed the data, assisted in drafting the manuscript, and approved the final version to be published. TM assisted in creating data collection tools, performed data collection through imaging review, analyzed the data, assisted in drafting the manuscript, and approved the final version to be published.

## Funding

This study was supported by the Department of Anesthesiology and Critical Care (NIH Core Grant P30 CA008748).

## Declaration of competing interest

The authors declare no conflicts of interest in relation to this article. Dr. Amitabh Gulati is a consultant for AIS Healthcare, Medtronic, Flowonix, SPR Therapeutics, Nalu Medical, Tremeau Health, Hinge Health and Spark medical.

## Figures and Tables

**Table:** 

		Combined	Left	Right	Male Combined	Female Combined	BMI<25 Combined	BMI 25–30 Combined	BMI>30 Combined	Age<70 Combined	Age>70 Combined
S1	Average Angle	24.58960938	24.96242188	24.21679688	23.35535714	26.07922414	26.0591	25.80128315	22.12964286	24.90844828	24.32542857
	STD	7.892873622	7.690564994	8.101302987	7.694608151	8.548159104	10.64486385	6.851195861	8.460614887	10.37574067	7.826726663
	Margin of Error	1.367370593	1.884188424	1.984819232	1.802575897	2.199959428	2.950605721	2.53771846	2.558782391	2.670306932	1.833526614
	Upper bound	25.95697997	26.8466103	26.20161611	25.15793304	28.27918357	29.00970572	28.33900161	24.68842525	27.57875521	26.15895519
	Lower Bound	23.22223878	23.07823345	22.23197764	21.55278125	23.87926471	23.10849428	23.26356469	19.57086047	22.23814134	22.49190196
S2	Average Angle	33.71937008	32.68404762	34.73851563	32.96137681	34.62112069	33.8811	32.0791181	33.74214286	33.46631579	33.92542857
	STD	11.10705299	11.00669028	11.18092523	10.36765845	12.81192709	13.42215065	11.64193172	12.26199478	13.1968256	10.9322905
	Margin of Error	1.931759491	2.717956755	2.739326683	2.446314038	3.29728535	3.720430348	4.391361978	3.70845107	3.426006013	2.561050929
	Upper bound	35.65112957	35.40200437	37.47784231	35.40769085	37.91840604	37.60153035	36.47048008	37.45059393	36.8923218	36.4864795
	Lower Bound	31.78761059	29.96609086	31.99918894	30.51506277	31.32383534	30.16066965	27.68775612	30.03369179	30.04030978	31.36437764
SIJ	Average Angle	33.04800781	32.65992188	33.43609375	32.45778571	33.76034483	32.1089	32.55374167	33.37035714	32.54655172	33.4635
	STD	11.27779304	11.88250588	10.70765949	11.02337742	12.58160273	13.44834324	12.47544525	10.8433213	13.27057321	11.54608638
	Margin of Error	1.953777965	2.911213941	2.623376575	2.582389388	3.238008933	3.727690562	4.620969589	3.279395172	3.415322795	2.704841703
	Upper bound	35.00178578	35.57113582	36.05947032	35.0401751	36.99835376	35.83659056	37.17471126	36.64975231	35.96187452	36.1683417
	Lower Bound	31.09422985	29.74870793	30.81271718	29.87539633	30.52233589	28.38120944	27.93277208	30.09096197	29.13122893	30.7586583
